# Recent Advances of Photoactive Near-Infrared Carbon Dots in Cancer Photodynamic Therapy

**DOI:** 10.3390/pharmaceutics15030760

**Published:** 2023-02-24

**Authors:** Jinxing Song, Xiaobo Gao, Mei Yang, Weiju Hao, Ding-Kun Ji

**Affiliations:** 1 Institute of Molecular Medicine, Renji Hospital, Shanghai Jiao Tong University School of Medicine, Shanghai 200127, China; 2 Department of Anatomy and Physiology, Shanghai Jiao Tong University School of Medicine, Shanghai 200240, China; 3 School of Materials and Chemistry, University of Shanghai for Science and Technology, Shanghai 200093, China

**Keywords:** carbon materials, NIR carbon dots, photosensitizer, photodynamic therapy, cancer treatment

## Abstract

Photodynamic therapy (PDT) is a treatment that employs exogenously produced reactive oxygen species (ROS) to kill cancer cells. ROS are generated from the interaction of excited-state photosensitizers (PSs) or photosensitizing agents with molecular oxygen. Novel PSs with high ROS generation efficiency is essential and highly required for cancer photodynamic therapy. Carbon dots (CDs), the rising star of carbon-based nanomaterial family, have shown great potential in cancer PDT benefiting from their excellent photoactivity, luminescence properties, low price, and biocompatibility. In recent years, photoactive near-infrared CDs (PNCDs) have attracted increasing interest in this field due to their deep therapeutic tissue penetration, superior imaging performance, excellent photoactivity, and photostability. In this review, we review recent progress in the designs, fabrication, and applications of PNCDs in cancer PDT. We also provide insights of future directions in accelerating the clinical progress of PNCDs.

## 1. Introduction

Cancer is still a worldwide health issue, causing nearly 10 million deaths annually [1]. Traditional cancer treatments in clinics, including chemotherapy, radiotherapy, and surgery, commonly lead to many side effects, including immune system imbalance, drug resistance, or postsurgical wound infections, which affect greatly the effectiveness of cure and rehabilitation of patients [2,3]. New approaches with both a high therapeutic efficacy and low side effects are highly needed and have been attracting tremendous attention. Photodynamic therapy (PDT), as a promising cancer treatment, has attracted continuous attention and developed rapidly in recent years [4]. It has a non-invasive nature with low toxicity, no drug resistance, and minimal side effects. Furthermore, PDT can be combined with other therapy methods, e.g., photothermal therapy (PTT), chemotherapy, and immunotherapy, to minimize the potential side effects and enhance the opportunity for patients to be cured [5].

**Figure 1:**
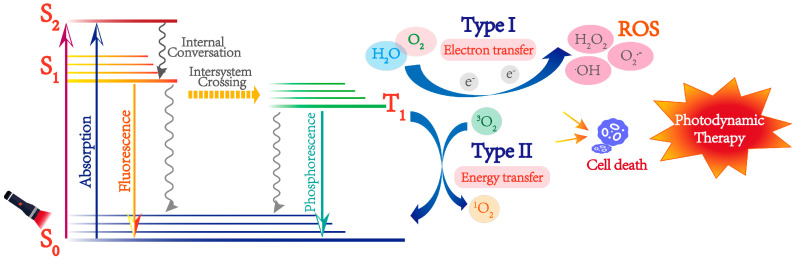
Schematic illustration of the modified Jablonski diagram of a PS in PDT [14].

The realization of photodynamic treatment includes three important factors; PS, light, and oxygen [6]. Among them, PSs directly affect the effect of photodynamic treatment. The PS enriched in tumor sites can be activated under the light of a specific absorption wavelength. The generated reactive oxygen species (ROS) could induce the apoptosis and necrosis of the tumor cells [7]. Figure 1 shows the underlying mechanisms of PDT. When PSs are exposed to light irradiation with proper wavelength, electrons of PS molecules transit from the ground state (S_0_) to the excited singlet (S_1_), and some of the singlet PS molecules jump to the triple state (T_1_), splitting sensitive molecules into two mechanisms when producing reactive oxygen species. At the triple excited state, PSs can divide into two types: type I and type II. One is an electron or proton transfer with adjacent substrates and molecular oxygen to produce superoxide negative ions (O_2_-), further forming cytotoxic ROS such as hydroxyl radicals and hydrogen peroxide (H_2_O_2_) (Type I) [7]. The other is based on triplet-triplet annihilation, in which the PS is excited at its triplet state and directly reacts with natural existed molecular oxygen (^3^O_2_), and the cytotoxic singlet oxygen molecules (^1^O_2_) are generated through the electron energy transfer in this state (Type II) [8]. The Type II PSs are more oxygen concentration-dependent, and many studies are also focusing on the PSs that can better produce reactive oxygen species through the Type I pathway. In addition to the influence of oxygen concentration, the absorption of tissue penetration of PSs is also another important factor affecting the treatment.

Thus far, clinical photodynamic therapy is widely used for skin diseases or superficial tumors. Visible light irradiation is the most popular method for photodynamic therapy. However, visible light displays poor penetration ability in human tissues, which limits its cancer treatment effects [9]. Thence, a number of NIR organic PSs have been developed and used for cancer PDT due to their high ^1^O_2_ generation quantum yield (QY) and tissue penetration, such as porphyrin [10], hypocrellin [11], and phthalocyanine [12]. However, such NIR organic PSs have the disadvantages of low water solubility, easy aggregation, and poor tumor-targeting capability, resulting in a series of side effects [13]. Therefore, developing novel PSs to overcome the above limitations is essential for the PDT of cancer.

Carbon dots (CDs) are a new type of carbon nanomaterials, with less than 10 nm in size, including carbonized quantum dots, graphene quantum dots, carbon nanodots, and carbonized polymer dots [15,16,17,18,19]. The carbon dots have a π-π conjugate structure, which is significantly different from the traditional inorganic quantum dots. Their core is composed of graphitized sp^2^ hybrid carbon, and the shell is rich in organic functional groups, such as carboxyl group, aminyl group, hydroxyl group, etc., making the carbon dots easily subjected to a variety of surface modifications [20]. In addition, compared with traditional organic fluorescent dyes, carbon dots have excellent photobleaching resistance, low toxicity, are water-soluble, and do not contain toxic metal elements, with good biocompatibility [21,22]. With the improvement of the CDs preparation method, the CDs have developed from the original blue-green light to the near-infrared CDs (NCDs). More importantly, many CDs have the ability to convert light energy into ROS and therefore be used as PSs for cancer PDT [23,24,25,26,27]. In particular, NCDs with an absorption wavelength above 650 nm have deeper tissue penetration, exhibiting good photodynamic effects to be used as photodynamic therapeutic PSs [28].

**Figure 2:**
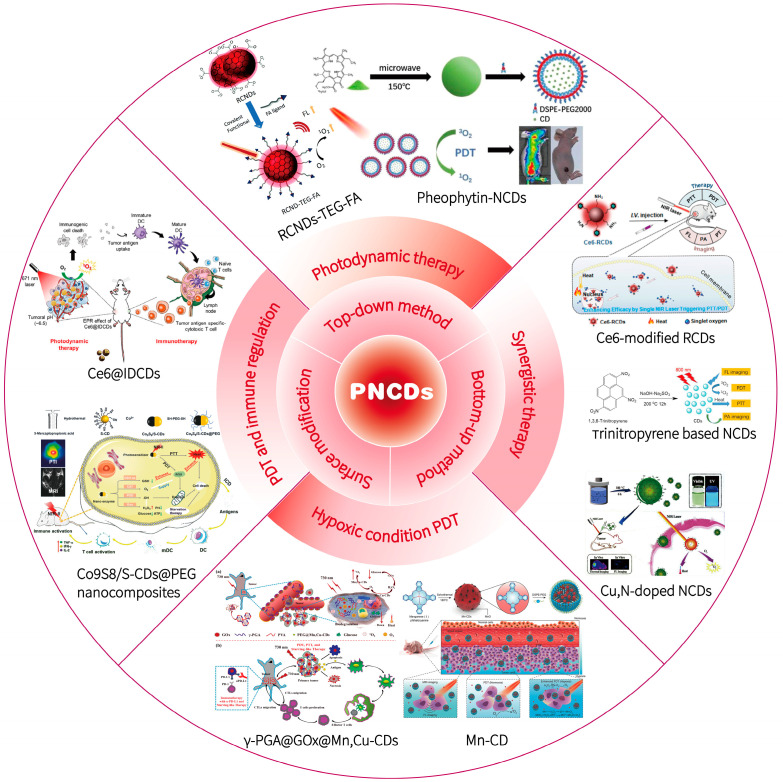
Schematic illustration of PNCDs preparation and PDT related application.

Here, we review recent progress in the design, fabrication, and application of PNCDs in cancer photodynamic therapy (Figure 2). We begin by introducing various methods for the design and preparation of PNCDs. Then, recent applications of PNCDs in cancer photodynamic therapy are highlighted. Finally, we offer an insight into the challenges and future perspectives of PNCDs in cancer photodynamic therapy.

## 2. Preparation of PNCDs

To produce PNCDs, both appropriate synthetic conditions and starting well-defined precursors are important, which can afford functional CDs with controlled surface chemistry, tunable optical properties, and microscopic morphology [15]. Multifarious precursors, containing animal derivatives, vegetables, and some other common reagents have been utilized for the preparation of CDs [29]. Recently, by carefully choosing proper precursors, a number of PNCDs have been reported, and the reported approaches mainly consist of three categories: the top down method, the bottom up method, and surface modification.

### 2.1. Top-Down Method

The top-down method, namely, cutting bulk carbon materials such as carbon nanotubes [30], graphite [31,32], or fibers [33] into small fragments (<10 nm) is usually via discharge, laser ablation, strong acid oxidative cracking, and electrochemical oxidation. Considering the harsh reaction condition, and energy consumption, HNO_3_ or H_2_SO_4_ is often used in the process and introduces nitrogen or sulfur elements into the CDs, increasing the surface defects to form surface energy potential and adjusting the optical properties [34]. As for this route to prepare prolonged wavelength CDs, further treatment was carried out by reacting with NaBH_4_ or NaOH to balance the carbonyl group and π-electron system, resulting in a red shift of CDs [35]. Additionally, increasing the size of CDs can lead to a red-shift of emission and absorption wavelengths [32].

In 2013, Lee et al. [36] reported a simple synthesis of near-infrared graphene nanodots (above 700 nm in wavelength). They used a mixture of strong acids (sulfuric acid and nitric acid) to break down the planar structure of graphene. By controlling the reaction temperature, they found that the emission wavelengths of the graphene nanodots varied from 460 nm to 805 nm and their absorption wavelengths range from 200 nm to 800 nm. However, their NIR photoactivity was not tested. Recently, some polythiophene derivatives were used as carbon sources to prepare photoactive CDs [26,37,38]. In 2020, Ji et al. [38] prepared a novel PNCD by hydrothermal treatment using polythiophene as the precursor. After heating with NaOH at 200 °C for 10 h, the PNCDs were obtained with an emission wavelength ranging from 500 to 800 nm and an absorption wavelength ranges from 200 to 700 nm. After modification with folic acid (FA), the resulting PNCDs displayed a high ^1^O_2_ yield of 0.4 in water under the irradiation of a 660 nm laser.

To date, PNCDs have been successfully prepared via the top-down method by some groups. However, the strong acid and alkali destroy the π-conjugated structure in CDs, which could lead to short absorption and emission wavelengths. Thus, carbon source, reaction time, and temperature should be well designed and controlled. It is still a challenge for mass production of PNCDs using the top-down method.

### 2.2. Bottom-Up Method

In the bottom-up method, small molecules or polymers are fused chemically through dehydration or assembly and then condensation or polymerization to obtain certain size CDs with different optical properties [39]. Additionally, many natural animal or vegetable derivatives can be used as precursors to prepare CDs [17]. Generally, as the rich carbon sources for bottom-up preparation, hydrothermal/solvothermal method [25,38,40,41,42,43,44,45,46,47,48,49,50], microwave synthesis [51], microwave-assisted hydrothermal method [52], and solvent-free carbonization [53] are commonly used to treat precursors. Compared to the top-down method, bottom-up treatment has relatively mild conditions and richer sources of raw materials. This route has gradually become the primary approach to prepare CDs with different emission bands.

Many photoactive small molecule compounds, such as methylene blue [54], porphyrin derivatives [51,55], manganese (II) phthalocyanine [51], were chosen as carbon sources to prepare PNCDs. In addition to small molecules, natural polymers and biomass are also favorable carbon sources for PNCDs. In 2021, NIR emission CDs originated from spinach were prepared by Liu et al. [56]. The biomass CDs were bound with chlorophyll and copper ions on the surface, leading to reduced energy level difference within the chlorophyll molecules. Further ROS was generated under 660 nm laser irradiation. In addition, free biothiol in cancer cells was bound with the CDs surface, which can enhance PDT. Recently, Kang and his colleagues [57] synthesized Co_9_S_8_/S-CDs@PEG (CSCs@PEG) nanocomposites by a two-step hydrothermal treatment. Firstly, sulfur-CDs were prepared with 3-mercaptopropionic acid as a precursor in an equal ratio of water and ethanol and the -SH was distributed on surface of the CDs. Then, cobalt nitrate hexahydrate was mixed with the CDs and reacted at 200 °C for 12 h to form the composite. The CSC@PEG showed NIR-II emission and absorption wavelengths (higher than 1000 nm), and possesses a high-level ROS generation capability under a 1064 nm laser.

### 2.3. Surface Modification

In addition to directly preparing PNCDs, the abundant functional groups on the surface of CDs such as -COOH, -NH_2,_ and -SH make them proper platforms for further modification to meet requirements in PDT. The poor water solubility, aggregation, and hydrophobic interaction of traditional small molecule PSs such as porphyrin and its derivatives dramatically limit their application in clinics. CDs with good biocompatibility and water solubility can be excellent carriers for PSs, either by chemical grafting or non-covalent assembly. To date, many NIR PSs, such as Ce6 [58,59], protoporphyrin [60], Aminoporphyrins [61], and Protoporphyrin IX [62] have been integrated with water-soluble CDs, resulting in a number of NCDs with high biodispersibility and NIR photosensitivity.

Recently, Santos et al. [61] synthesized graphene-based CDs (GQDs) linked with porphyrins via amide bond. The GQDs were prepared via the hydrothermal method of graphene oxide. Then, amino-porphyrins were modified on the GQDs surface via amide linkage using thionyl chloride (SOCl_2_) and 1-ethyl-3-(3′-dimethylaminopropyl)carbodiimide coupling methodologies. The resulting GQDs-Porphyrinsconjugates displayed better stability than porphyrin in biological media. An in vitro experiment indicated GQDs-Porphyrins conjugates its own negligible dark cytotoxicity. The IC50 of them ranges from 1–10 nM, 10 times lower than that of porphyrin. However, the overall PDT efficiency of the two conjugates was similar to porphyrin, which contributed to the balance of an effective porphyrin cell uptake and a reduced ^1^O_2_ generation of porphyrin conjugates. Generally, grafting small molecule PSs is to endow CDs with new biomedical properties and meanwhile share the excellent nature of CDs, thus achieving an enhanced therapeutic effect. Sun et al. [63] modified CDs with 0.56% mass of Ce6 via amide condensation. As a result, the Ce6-RCDs presented as more photostable than free Ce6 and realized enhanced photodynamic and photothermal therapy.

Additionally, upconversion CDs is one kind of novel PNCDs. It can work as NIR PSs carrier to absorb long-wavelength light and then emit short-wavelength light. The emitted light could excite the carried organic PSs by FRET mechanism to produce ROS. Recently, Wang et al. [64] prepared 808 nm near-infrared light-triggered upconversion CDs. The upconversion CDs act as tremendous “antennas” to efficiently absorb an NIR laser, and the tight coupling between the CDs and porphyrin in MOFs greatly shortened the distance between the two components and improved the efficiency of the energy transfer from the CDs to porphyrinic MOFs, thus yielding a sharply enhanced ^1^O_2_ generation capability with 808 nm laser irradiation.

## 3. Application of PNCDs in Cancer PDT

Compared with common small molecule PSs, PNCDs have excellent water solubility, biocompatibility, and especially deep tissue penetration, allowing them to realize PDT therapy from superficial parts to deep tissue [65]. To avoid the insufficiency of monotherapy and increase cancer treatment effect, combination therapies with clinical treatment strategies, including chemotherapy, radiotherapy, and immunity therapy, have been developed.

### 3.1. PNCDs for Cancer PDT

**Figure 3:**
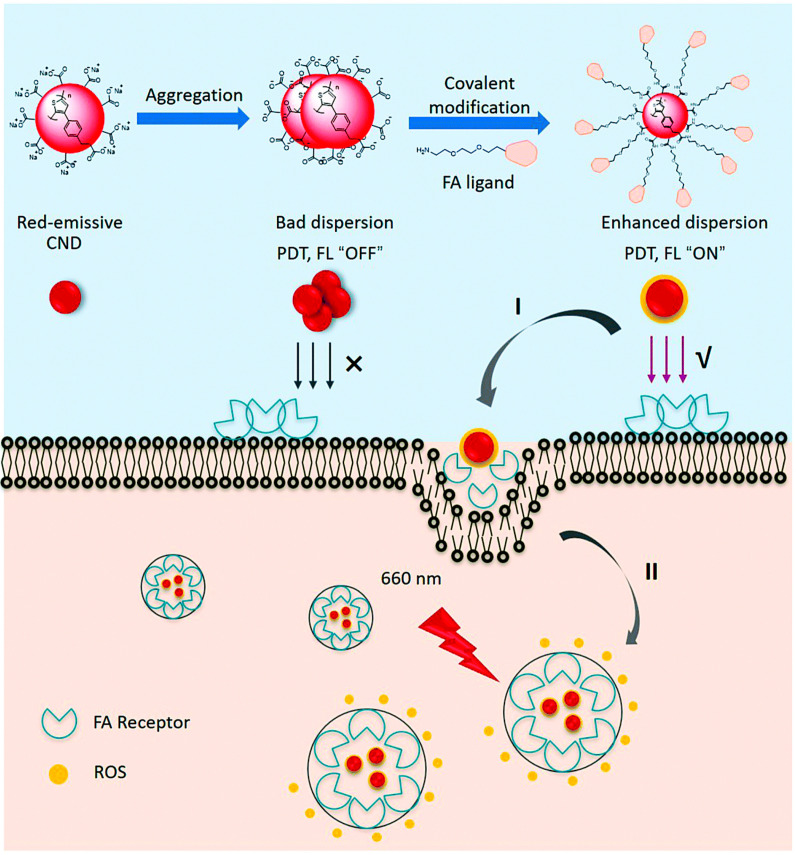
Schematic illustration of the covalent functionalization of RCNDs and their targeted intracellular production of ROS through (**I**) FR-mediated endocytosis and (**II**) light irradiation. FL stands for fluorescence, FA stands for folic acid [38]. Copyright 2020, Royal Society of Chemistry.

With the outstanding advantage for tissue penetration, excellent water solubility and compatibility, PNCDs to generate ROS are promising PSs for an enhanced PDT effect to compared with small molecule PSs. These PNCDs also exhibited fluorescent properties for bioimaging to assist the PDT process. Ji et al. [38]. prepared a red emission carbon dot through a hydrothermal method using polythiophene phenylpropionic acid as carbon source (Figure 3). After controlled modification with folic acid ligand, the resulting RCNDs-TEG-FA exhibited excellent water solubility compared to primitive RCNDs and also a stable fluorescent signal, even after 24 h incubation. Importantly, there was a strong intracellular ROS generation with a low-power laser at 660 nm of 0.1 W cm^−2^, indicating a great potential for PDT. Further validation in Hela cells showed good compatibility of the RCNDs-TEG-FA with a concentration range of 0–100 μg mL^−1^, while as the laser irradiation began, a mortality rate of 70% of the cells was observed at 100 μg mL^−1^, confirming a sufficient amount of generation of intracellular ROS and efficient tumor cell death.

PNCDs with NIR emission wavelength are always attracting tremendous attention. Wen et al. [51] prepared hydrophobic CDs with a maximum NIR emission peak at about 680 nm with pheophytin as a precursor. Under 671 nm laser irradiation, the CDs show a high generation of ^1^O_2_ with a quantum yield of 0.62, exhibiting an increased ROS amount with laser irradiation. The CDs exhibited a high 4T1 cell viability of over 90% in dark and about 95% mortality after laser irradiating (671 nm, 0.1 W cm^−2^ for 10 min) at the concentration of 250 μg mL^−1^. During in vivo experiments, the CDs showed tumor site enrichment compared to other organs and low toxicity. Fourteen-day photodynamic treatment destroyed the tumor cells in 4T1 tumor-bearing mice, proving an effective generation of ROS produced by the NCDs and achieving PDT in vivo.

To increase tissue penetration, Wang et al. [66] designed the energy transfer CDs hybrid system by modifying GQD on the surface of upconversion nanoparticles (UCNPs) to form a GUCNP nanosystem. In this nanosystem, fluorescence resonance energy transfer (FRET) from UCNPs to GQDs could significantly facilitate the NIR fluorescence enhancement and NIR light-activated ^1^O_2_ generation. Upon 980 nm laser irradiation, the ^1^O_2_ probe showed a strong fluorescence but no significant fluorescence without irradiation, indicating a considerable ^1^O_2_ generation. Additionally, the CDs showed 38.4% of the late apoptotic cells in vitro. In vivo data indicated that the CDs can inhibit 4T1 tumor growth dramatically under a 980 nm laser irradiation. No noticeable pathological changes were observed in tumor tissues without laser irradiation.

### 3.2. PNCDs for Synergistic PDT and PTT

Though the excellent PDT effects of PNCDs and their potential for deeper tissue tumors therapy, some limitations, including low drug enrichment efficiency, and penetration, could reduce the effects of cancer PDT [67]. Under the premise of not increasing the PNCDs amount, maintaining an enhanced therapeutic effect is very important. Similar to PDT, PTT is another noninvasive therapeutic strategy. PTT employs photosensitizers to convert photon energy into heat. The light-induced hyperthermia is able to effectively kill cancer cells. PTT requires laser irradiation with reasonable power to generate heat, and the PTT effect highly depends on the photothermal conversion efficiency (η%) of the CDs [68,69]. As PDT and PTT have their individual limitations, a combination of the two invasive therapies provides inspiration [70].

**Figure 4:**
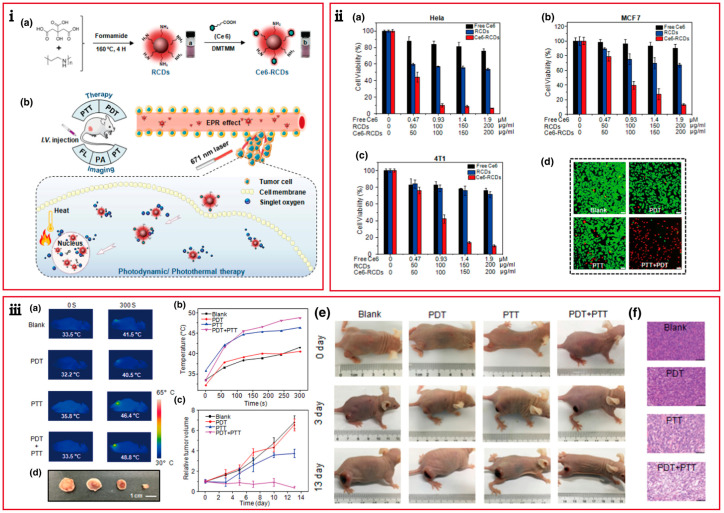
Preparation and PDT in vitro and in vivo of Ce6-RCDs, (**i**) Research schematic diagram of the Ce6-RCDs, (**a**) Schematic diagram of the preparation of the Ce6-RCDs, (b) Schematic illustration of imaging and synergistic PTT/PDT process in vivo. (**ii**) (**a**) HeLa, (**b**) MCF-7, and (**c**) 4T1 cell cytotoxicity treated by PNCDs of various concentrations, (**d**) Calcein-AM/PI co-staining fluorescence images of Hela cell lines for PDT/PTT estimation. (**iii**) In vivo PDT effect of the PNCDs. (**a**) Thermal images of tumor-bearing mice before and after laser irradiation, (**b**) Temperature elevation curves of tumor regions with laser irradiation time, (**c**) Tumor growth curve recorded from 0 to 13 days, (**d**) Digital photographs of tumors, (**e**) Digital photographs of tumor-bearing mice during the treatment, (**f**) H&E staining of tumors collected from the four group treatments after 1 day. The scale bar is 100 μm [63]. Copyright 2019, American Chemical Society.

Sun and his colleagues [63] prepared PDT/PTT amino-rich CDs and modified 0.56% mass of Ce6 to endow the CDs (Ce6-RCDs) with both photothermal therapy (PTT) and photodynamic therapy (PDT) properties (Figure 4). After a 671 nm laser irradiated for 15 min, the ^1^O_2_ sensor indicator showed ^1^O_2_ generation without being influenced by Ce6 modification. The CDs reduced the cytotoxicity of a single Ce6 exhibiting negligible cytotoxicity in Hela, MCF-7, and 4T1 cells. In vitro experiments indicated inadequate cell killing performance of either PDT (Ce6) or PTT (RCDs) group. While the cell viability decreased dramatically with increasing Ce6-RCDs concentration under laser irradiation (671 nm, 500 mW cm^−2^), nearly all tumor cells were destroyed, validating the enhanced anti-tumor effect. Further animal models showed a similar trend of a therapeutic effect as in vitro.

Lan et al. [47] designed a kind of multifunctional two-photon excited PNCDs for bioimaging and synergistic PDT/PTT in cancer treatment. The PNCDs could generate ^1^O_2_ and showed excellent photothermal conversion capability with a single NIR laser irradiation at 800 nm. In in vitro photodynamic treatment, the cell viability of Hela cells was about 100% after incubating with CDs at concentrations up to 100 μg mL^−1^. With 800 nm laser irradiation, the cell viability was reduced to 10%. Then, the in vivo PDT demonstrated that the CDs under irradiation exhibited an efficient inhibition of a 4T1 murine breast tumor.

Guo et al. [49] designed a novel Cu, N-doped CDs (Cu, N-CDs) with an absorption peak at around 740 nm and under the irradiation of an 808 nm laser (1.0 W cm^−2^), the temperature changed from low to high, rising from 25 to 54 °C in 10 min. The B16 cell viability sharply decreased from 88% to 20%, confirming the in vitro PDT effect, and simultaneously ^1^O_2_ is generated for PDT. In in vivo therapy, the Cu, N-CDs displayed an evidently inhibited tumor growth in B16 melanoma tumors bearing mice. Noticeably, there was no obvious inhibition either solely laser irradiation or Cu, N-CDs injection. Doping metal ions into the NIR CDs actually enhanced NIR absorption of the Cu, N-CDs and then benefited the synergistic PDT/PTT.

**Figure 5:**
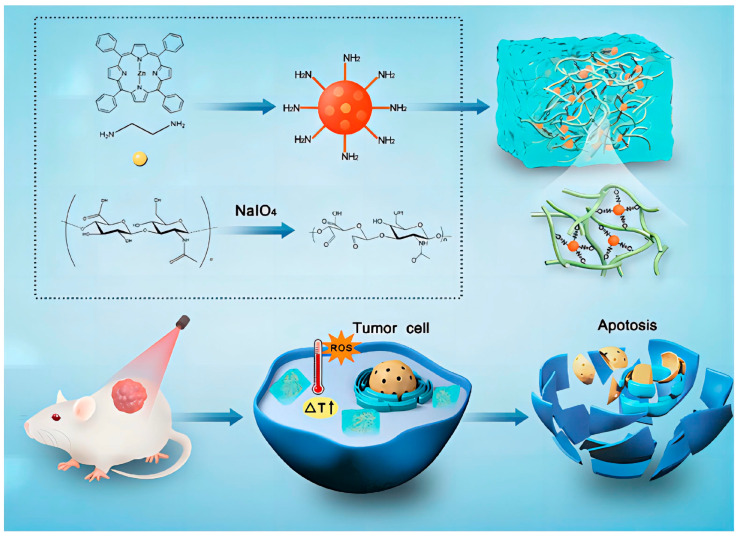
Schematic Diagram of the Synthesis Process of CD@Hydrogel and Its Application in Cancer Treatment [40]. Copyright 2022, American Chemical Society.

Recently, the construction of a PDT system including photosensitizer and hydrogel has aroused great interest in tumor therapy. Yue et al. [40] prepared an injectable hydrogel based on the Schiff base reaction between HA-CHO and carbon dots, which can realize PTT and PTT simultaneously (Figure 5). In this hydrogel, CDs with rich −NH_2_ can be used not only as a photosensitizer but also as an efficient crosslinking agent for the Schiff base reaction to form a hydrogel network. The CD@Hydrogel with good biosafety showed a high antitumor effect after 660 nm laser irradiation in in vitro and in vivo experiments.

### 3.3. PNCDs for Hypoxic PDT

PNCDs can efficiently generate ^1^O_2_ for cancer PDT. However, the hypoxic tumor microenvironment and rapid consumption of oxygen in the PDT process will severely limit therapeutic effects of PNCDs due to the oxygen-dependent PDT [48]. Thus, it is becoming particularly important to develop novel PNCDs as an in situ tumor oxygenator for overcoming hypoxia and substantially enhancing the PDT efficacy.

**Figure 6:**
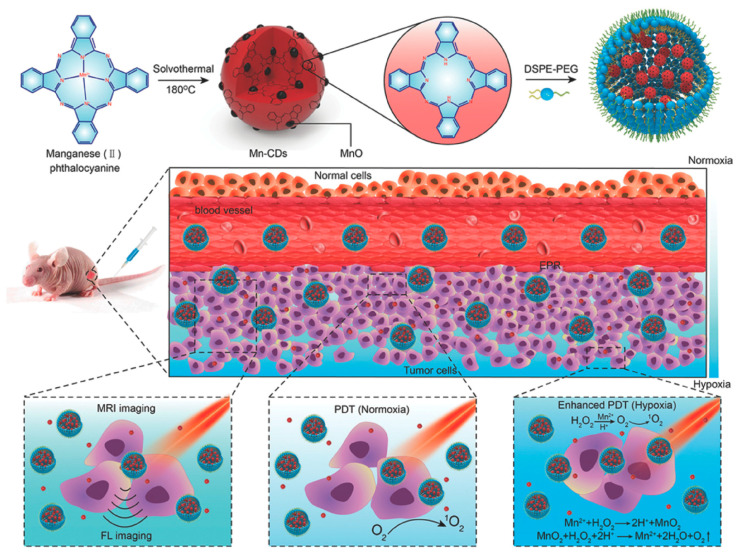
Schematic illustration of the Mn-CD assembly as an acidic H_2_O_2_-driven oxygenator to enhance the anticancer efficiency of PDT in a solid tumor [48]. Copyright 2018, Wiley.

In 2018, Zhang et al. [48] successfully prepared magnetofluorescent Mn-CDs using manganese(II) phthalocyanine as a precursor. After cooperative self-assembly with DSPE-PEG, the obtained Mn-CD assembly can be applied as a smart contrast agent for both near-infrared fluorescence and T1-weighted magnetic resonance (MR) imaging (Figure 6). The as-prepared Mn-CD assembly can not only effectively produce ^1^O_2_ with a quantum yield of 0.40, but also highly catalyze H_2_O_2_ to generate oxygen. The Mn-CD assembly can be utilized as an acidic H_2_O_2_-driven oxygenator to increase the oxygen concentration in hypoxic solid tumors for simultaneous bimodal FL/MR imaging and enhanced PDT.

Recently, Zhang et al. [26] prepared three types of PNCDs with maximum emission at approximately 680 nm. They exhibit adjustable ROS production with equal superoxide anion (via type I PDT) and incremental ^1^O_2_ (via type II PDT). NCDs enable themselves to induce cell programmed death via activating mitochondrion-mediated apoptotic pathways. This work exploits the unprecedented NCDs with tunable type I and type II ROS generation that could ensure highly efficient tumor eradication both in vitro and in vivo, even under the harsh tumor microenvironment.

### 3.4. PNCDs for Synergistic PDT/PTT/Immunotherapy

In the last few decades, immunotherapy has received much attention and opened a new window for cancer treatment. Immunotherapy utilizes our own immune system’s both innate and adaptive immune response to fight against cancer cells. Importantly, it can realize precise targets and long-lasting attacks, forming immune memory [71,72,73,74]. However, the poor immunogenicity of tumor tissues brings difficulty to clinical application [75]. Proper immune regulation or activation is important. Some PNCDs can activate an immune response in tumors and perform PDT/PTT simultaneously, regulating tumor immune response [76,77], and indicating a further developing direction of PNCDs for PDT.

Kim and his colleagues [76] developed pH-sensitive CDs (Ce6@IDCDs) modified with Ce6. Ce6 was released at tumoral pH 6.5, causing immunogenic cell death through the PDT process under a 671 nm laser irradiation. The CDs treated cells led to an increased expression level of CD80 and CD86, which indicated the maturation of the dendritic cells (DCs). In vivo PDT obtained as consistent results as in vitro, and the CDs displayed excellent antitumor effects comparing the tumor volumes. The percentage of activated NK cells increased as well as CD4^+^ and CD8^+^ T cells. IL-2 and IFN-γ secretion in the Ce6@IDCD-treated group also indicated a successfully induced immune activation during the PDT process.

**Figure 7:**
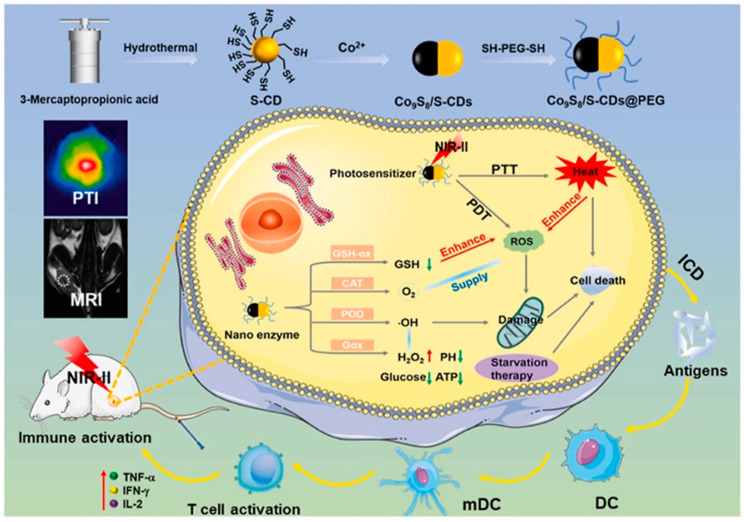
Schematic diagram of CSC@PEG for PTT/PDT/CDT starvation for anticancer [57]. Copyright 2022, Elsevier.

Additionally, Kang et al. [57] synthesized Co_9_S_8_/S-CDs@PEG (CSCs@PEG) nanocomposites with a NIR-II excited PDT and PTT upon irradiation of 1064 nm (1.16 eV) (Figure 7). The CSCs@PEG increased the temperature by 26.5 °C within 10 min (1064 nm, 0.8 W cm^−2^) and generated ROS including OH and ^·^O_2_^−^. Interestingly, the CDs exhibited selective cytotoxicity toward 4T1 tumor cells (cell viability of 56.18 ± 2.61%, 300 μg mL^−1^) and L929 cells (cell viability of 92.40 ± 2.82%, 300 μg mL^−1^), as the H_2_O_2_ overexpressed in tumor ensured the anticancer activity. Significantly, the CDs not only inhibited tumors in the PDT/PTT synergistic way, but also acted as tumor immunomodulators. DCs maturation induced by immunogenic death can further trigger an antitumor immune response. DC-maturated indicators (CD80^+^ and CD86^+^) confirmed a larger amount of mature DCs (29.8%) compared with control group of 8.6%. Related cytokines of immune regulation, such as TNF-α, IL-2 and IFN-γ, were all detected in obviously increased levels in the CSCs@PEG group. The CSCs@PEG exhibited various cancer therapy with a synergistic effect of NIR-II PDT/PTT/CDT and activation of immune response.

**Figure 8:**
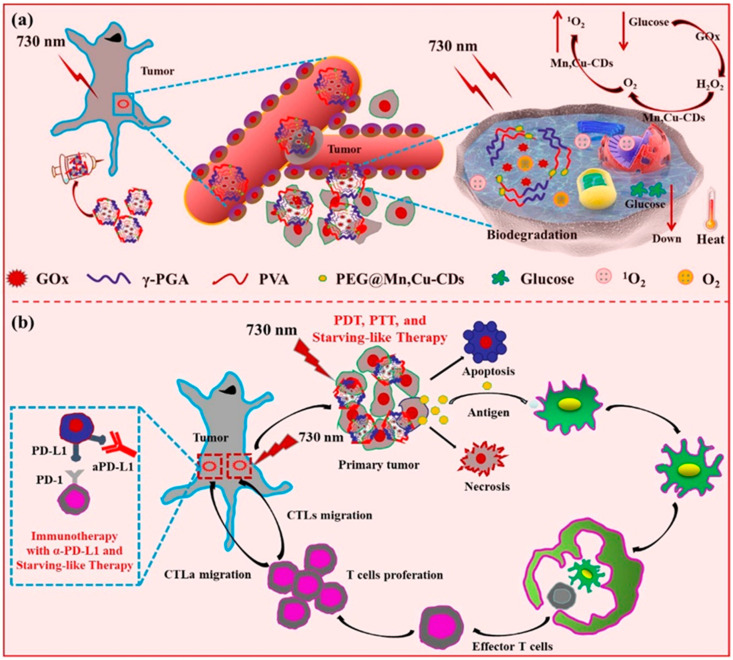
(**a**) Schematic illustration of starving and phototherapy mediated by γ-PGA@GOx@Mn, Cu-CDs NPs. (**b**) Schematic illustration of starving-like therapy, phototherapy, and immunotherapy mediated by γ-PGA@GOx@Mn, Cu-CDs NPs [77]. Copyright 2020, Elsevier.

To increase the amount of tumor antigens in the suppressive tumor microenvironment, Zhang et al. [77] developed a multifunctional nanoparticle γ-PGA@GOx@Mn, Cu-CDs (NPs). In this nanoparticle (Figure 8). Mn, Cu-doped carbon dots (CDs) worked as photosensitizers and self-supplied oxygenators. This multifunctional nanoparticle worked as a nanoreactor to induce the endogenous H_2_O_2_ generation and then to relieve hypoxia in tumors. Cancer-starving therapy is known for blocking nutrients’ supply to suppress tumor growth. The nanocomposites in this work reacted with glucose to form gluconic acid and H_2_O_2_, thus competing for nutrients with tumor cells, finally achieving a starving-like therapy. The NPs also displayed both photothermal and photodynamic effects under laser irradiation at 730 nm. The endogenous generation of hydrogen peroxide (H_2_O_2_) caused by the nanoreactors could significantly relieve tumor hypoxia and further enhance in vivo PDT. By synergistically combining the NPs-based starving-like therapy/PDT/PTT and check-point-blockade therapy, the cancer treatment efficiency was significantly improved.

## 4. Conclusions

In the last decade, PNCDs have attracted considerable attention in cancer photodynamic therapy due to their unique photophysical and photochemical properties, good biocompatibility, and deep tissue penetration. Although great progress has been achieved, biomedical applications of PNCDs in cancer photodynamic therapy are still in their early stages. Clinical applications remain difficult and challenging, and require considerable effort to perfect several aspects. (1) Standardization of PNCDs. It is widely accepted that the size, shape, and surface modification of PNCDs have an important influence on their toxicology and performance. However, it is still difficult to precisely control the size and shape of PNCDs with current preparation and purification technology. (2) Improving the therapeutic effect on deep tumors. For now, most of the current PNCDs are in the NIR I light region. They usually have weak absorption in the NIR photodynamic therapy window and no absorption in the NIR-II region, which hinders the effective treatment of deep tumors. (3) Biocompatibility of PNCDs. PNCDs display effective renal clearance due to their small size, leading to good biocompatibility and low toxicity. However, long-term toxicology evaluation in vivo is still needed to demonstrate the safety of PNCDs for further use in clinics. Although PNCDs still have many challenges in the clinical application of cancer PDT, we believe that the above challenges will be gradually solved with unremitting joint efforts of material scientists, chemists, physicists, biologists, and medical doctors.

## Data Availability

Data sharing not applicable.

## Abbreviations

**Full Name**	**Abbreviation**
Carbon dots	CDs
Fluorescence	FL
Fluorescence resonance energy transfer	FRET
Folic acid	FA
Fraphene-based CDs	GQDs
Magnetic resonance	MR
Near-infrared CDs	NCDs
Photoactive near-infrared CDs	PNCDs
Photodynamic therapy	PDT
Photosensitizers	PSs
Photothermal therapy	PTT
Quantum yield	QY
Reactive oxygen species	ROS
Red-emissive CNDs	RCNDs
Upconversion nanoparticles	UCNPs
